# Arthroscopic-assisted uni-portal ligament flavum sparing bone anchoring annular suture technique for lumbar disc herniation: A case report and literature review

**DOI:** 10.1097/MD.0000000000039763

**Published:** 2024-09-27

**Authors:** Gushang Xia, En Song, Qingli Kong, Xianglin Li

**Affiliations:** a Department of Orthopaedics, The People’s Hospital of Chuxiong Yi Autonomous Prefecture, Chuxiong, China; b Department of Sports Medicine, The First Affiliated Hospital of Kunming Medical University, Kunming, China.

**Keywords:** annular suture, arthroscopic-assisted uni-portal spinal surgery, bone anchoring technique, ligament flavum preservation, lumbar disc herniation

## Abstract

**Rationale:**

Lumbar disc herniation (LDH) manifests in diverse forms. If the nucleus pulposus or endplate tissues protrudes, the location of annular tears also varies, which poses various challenges for the annular suture technique. Tears at the annular attachment area at the edge of the vertebral body (cephalad or caudad) are considered a prohibitively challenging area for annular suturing.

**Patient concerns:**

A 37-year-old woman presented with a gradual onset of symptoms, experiencing leg pain and numbness over the left leg for 1 year before presentation. The pain radiated to the left S1 dermatome. Despite undergoing continuous medical therapy for more than 6 months, her symptoms showed no improvement. The strength of the left plantar flexion in the ankle and great toe was rated at 4 out of 5. The straight leg-raising and strengthening tests were positive for the left sides. Lumbar computed tomography and magnetic resonance imaging revealed left-sided disc herniation at the L5–S1 level, and nerve root compression, confirming the diagnosis of LDH.

**Diagnoses:**

The preoperative impression was LDH, intraoperative confirmation of a tear in the annular attachment area at the vertebral body edge.

**Interventions:**

This patient was treated with an arthroscopic-assisted uniportal spinal surgery (AUSS) technique focusing on ligament flavum (LF) preservation, protruding nucleus pulposus removal, nerve root decompression, and vertebral edge tear was then sutured using a bone anchoring annular suture with the Smile suture device.

**Outcomes:**

This technique was performed successfully in a patient with LDH. Significant improvements were observed in postoperative pain and numbness, Visual Analog Scale, and Japanese Orthopaedic Association scores. No postoperative instability or complications were observed, with computed tomography and magnetic resonance imaging confirming complete decompression. To the best of our knowledge, this is the first used AUSS with LF preservation and bone anchoring annular suture technique for LDH.

**Lessons:**

This case study demonstrates the AUSS combined with LF preservation and the bone anchoring annular suture technique provides favorable clinical and imaging outcomes and is a safe and effective technique for the treatment of LDH.

## 1. Introduction

LDH is a common and frequently-occurring disease in spinal surgery, constituting the most prevalent cause of radicular pain in the lower limbs.^[1]^ LDH prevalence has steadily increased in recent years, particularly among younger individuals, indicating a growing trend towards lower age of onset.^[2]^ LDH management primarily involves non-surgical approaches. Conservative treatment effectively alleviates symptoms in most patients with LDH, whereas approximately 25% may require surgical intervention due to an inadequate response to conservative measures or recurrent symptoms.^[3,4]^ Literature reports have confirmed that there is a recurrence rate of 5% to 19% after LDH surgery,^[5]^ and the reoperation rate is 4% to 15%.^[6]^ Preservation of the LF reduces the formation of epidural scars, enabling surgeons to locate anatomical structures and anatomical nerves.^[7,8]^ Reparation of annulus fibrosus tears after nucleus pulposus excision can significantly reduce LDH recurrence and reoperation rates.^[9]^ Additionally, it can diminish nerve root stimulation caused by inflammatory mediators released from the disc, relieve postoperative pain, and enhance postoperative quality of life.^[10]^ However, if the tear of the annulus fibrosus is situated in the attachment area of the vertebral body, it becomes a forbidden area for annulus fibrosus suture. Currently, the commonly employed surgical methods for the treatment of LDH include discoscopic technique, foraminoscope technique, microscope technique, unilateral biportal endoscopic technique, etc., all of which have demonstrated favorable outcomes. However, the literature available on LF preservation and suturing vertebral marginal annulus fibrosus tears associated with these aforementioned techniques is limited. In this study, we utilized a novel technique called AUSS, combining LF preservation with bone anchoring annulus suture for vertebral marginal annulus fibrosus tears, which has achieved satisfactory clinical results.

## 2. Case presentation

### 2.1. Ethics statement and case presentation

**Figure 1.**
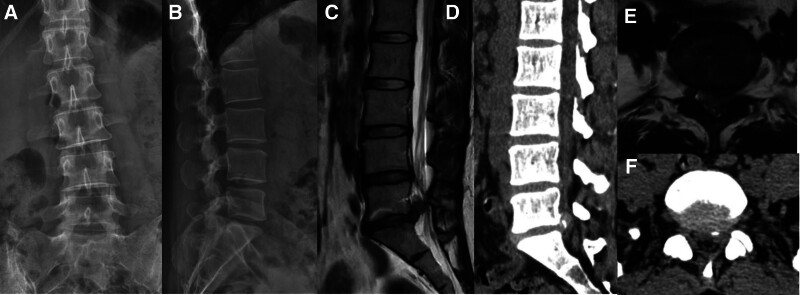
Patient preoperative radiologic images. (A, B) Preoperative anteroposterior and lateral radiographs. (C) Preoperative parasagittal T2-weighted magnetic resonance imaging (MRI) image. (D) Preoperative sagittal computed tomography (CT) image. (E) Preoperative axial T2-weighted MRI image. (F) Preoperative axial CT image.

This study was approved by the Institutional Review Board (IRB) of The People’s Hospital of Chuxiong Yi Autonomous Prefecture (IRB No. KYCS2024003). Written informed consent was obtained from the patient for the publication of any potentially identifiable images or data included in this article. A 37-year-old woman presented with a gradual onset of symptoms, experiencing leg pain and numbness over the left leg for 1 year prior to presentation. The pain radiated to the left S1 dermatome. She could not walk for over 20 minutes due to the pain. Her radicular leg pain in visual analogue scale was 7, and the Japanese Orthopaedic Association score was 12. Despite undergoing continuous medical therapy for more than 6 months, her symptoms showed no improvement. Neurological examination revealed no significant sensory abnormalities or apparent changes in bowel and bladder functions. The strength of the left plantar flexion in the ankle and great toe was rated at 4 out of 5. The straight leg-raising and strengthening tests were positive for the left sides. Physical examination revealed normal tendon reflexes in the bilateral lower limbs, and Babinski’s sign was negative. Flexion and extension lateral lumbar radiographs showed relative dynamic stability in all lumbar segments. CT showed no significant bony spinal stenosis or spondylolisthesis, and MRI revealed left-sided disc herniation at the L5-S1 level, and nerve root compression, confirming the diagnosis of LDH (Fig. 1).

### 2.2. *Procedure*

#### 2.2.1. Position, incision, and instruments

**Figure 2.**
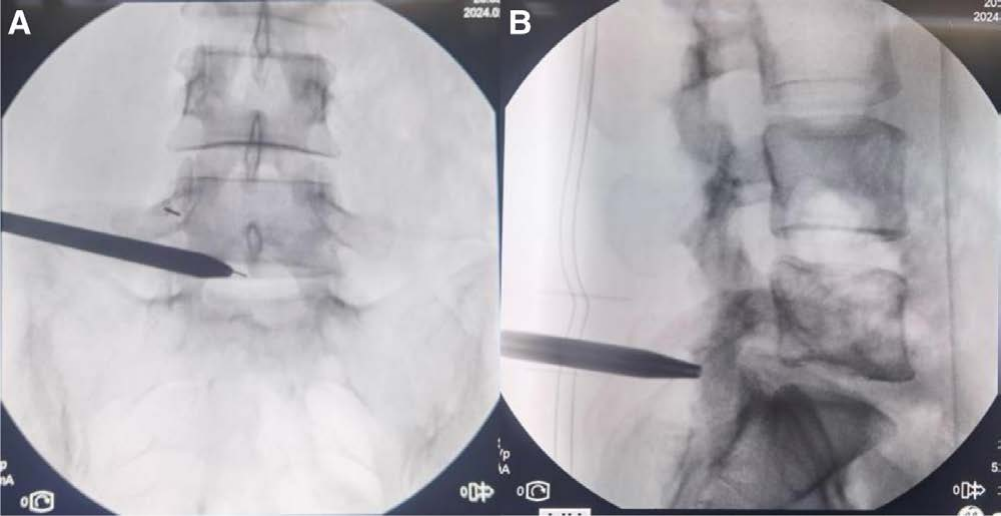
Intraoperative C-arm fluoroscopy used for localization. (A) Anteroposterior view demonstrates the precise positioning of the dilator at the junction between the spinous process and lamina. (B) Lateral view exhibits the parallel alignment of the dilator with respect to the intervertebral space.

The operation was performed under general anesthesia, with the patient positioned prone on an abdomen-free frame and the slightly flexed using a horseshoe headrest. C-arm fluoroscopy was employed for localizing the pathological level. The pedicles of L5 and S1 were selected as target positioning points, and the midpoint connection line between them was marked as the reference line. A longitudinal surgical incision of approximately 1.8 cm in length, parallel to the intervertebral space, was made along the reference line (Fig. 2). A 30° 3.0-mm diameter arthroscopes (Guangzhou Yunqi Medical, China), specially designed Smile annular suture instrument (Medical Technology Co., Ltd, China), high-definition imaging system, 3000-cc sodium chloride irrigation system, high-speed burr (Guizhou Zirui Technology, China), tool-kit of radiofrequency (RF) systems (Jiangsu BONSS Medical Technology, China), and open spine surgical instruments, including pituitary forceps, curettes, and Kerrison rongeurs, were used.

#### 2.2.2. AUSS for LF sparing discectomy

**Figure 3.**
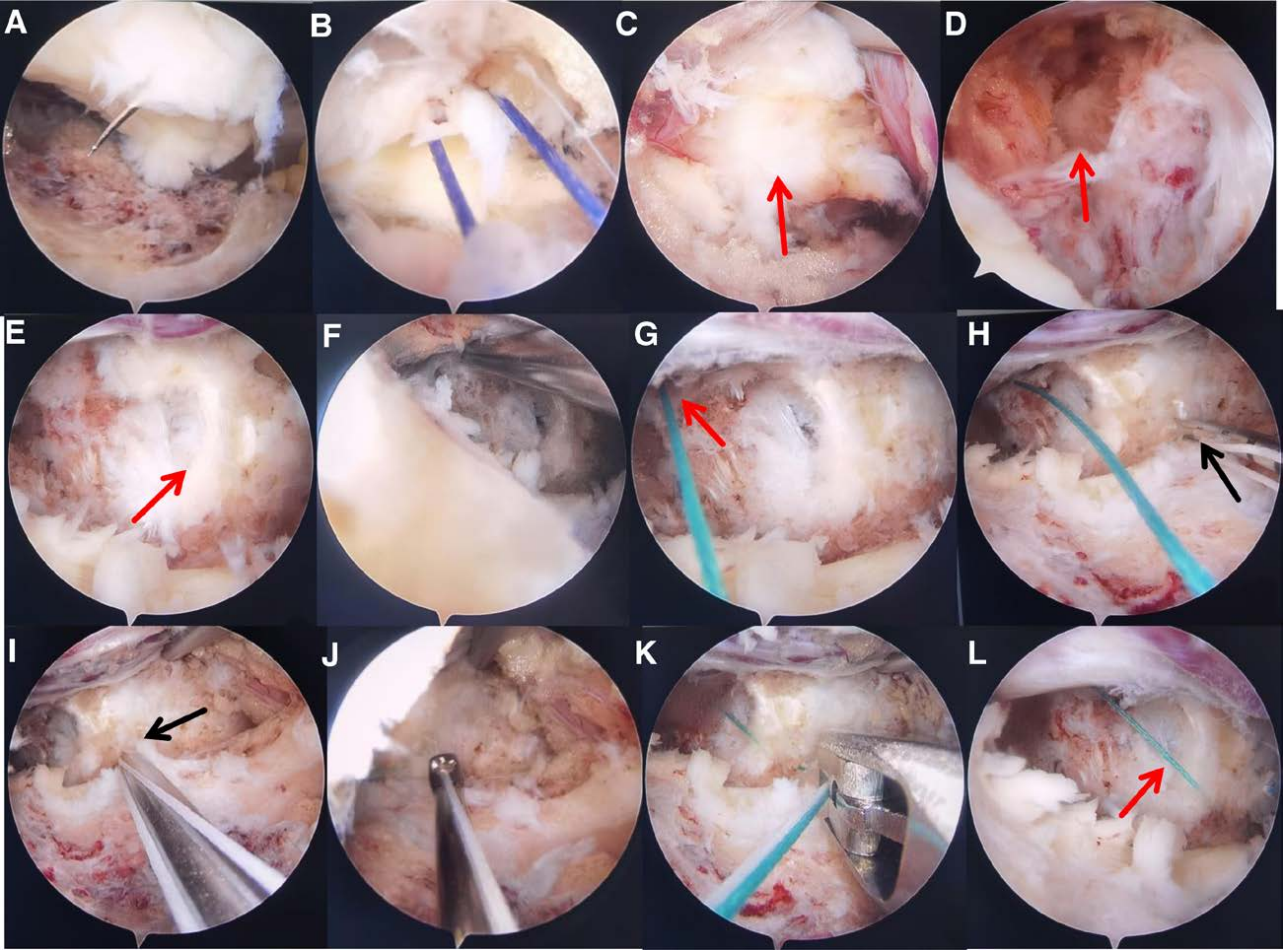
Operative illustrations in arthroscopic view. (A) A 4-0 absorbable thread was sutured with 1 stitch at the facet joint side of the flap-like ligament flavum. (B) Two threads were taken out of the incision to pull up the ligament flavum. (C) The protruding nucleus pulposus was fully exposed. (D) The damaged and protruding nucleus pulposus was removed, and the nerve root was thoroughly decompressed. (E) Arrow: a tear in the area where the annulus fibrosus attaches to the edge of the vertebral body. (F) A hole was created in the vertebral body with a Kirschner wire where located 4 mm from the edge of the tear. (G) The Smile suture needle was inserted into the vertebral body through the hole, the thread rod was pushed in, and the suture needle was pulled out. (H) Another suture needle with a coil attached was inserted into the seam sewn into the vertebral body, and subsequently, the needle was inserted into the annulus fibrosus side of the tear. (I) Arrow: the puncture point of the annulus fibrosus was 2 mm from the tear. (J) The knot was pushed in using a knot pusher. (K) The thread was cut using a suture cutter. (L) Arrow: the “bone anchoring” annular suture technique.

Firstly, a serial dilation instrument was inserted into the skin incision to facilitate gradual dilation and muscle peeling. The T-shaped peeler was introduced to ensure the complete removal of soft tissue from the lamina surface. An arthroscope was inserted and connected to a sodium chloride irrigation system for continuous gravity lavage. Soft tissues were dissected using RF, ensuring adequate hemostasis and pre-hemostasis to expose the junction area between the root of the spinous process and lamina of L5, as well as the medial edge of the facet joint and upper edge of the lamina of S1. The bone at the edge of the lamina was thinned using a high-speed burr employing the “circle method,” and the medial edge of the lamina and facet was bitten off using Kerrison rongeurs to reveal the proximal attachment edge, distal attachment edge, and facet joint side attachment edge of the LF. The LF was stripped using a nerve dissector to create a free petal-like structure with upper, lower and lateral edges. The facet joint side edge of the LF was sutured with a 4-0 absorbable silk thread without knotting. The double thread was extracted from the body, and a consistent traction force was maintained throughout the procedure to elevate the LF for intraspinal surgery, resembling the act of unveiling a “curtain,” known as the “ligament flavum suspension technique” (Fig. 3A,B). The RF and nerve dissector were meticulously employed to strip and preserve the epidural adipose tissue in a precise manner, ensuring careful exposure of the nerve root while separating any surrounding adherent tissue from the anterior portion of the dural sac. The protruding nucleus pulposus was fully exposed (Fig. 3C).Subsequently, the damaged and protruding nucleus pulposus was removed using pituitary forceps, the dural sac and nerve root were thoroughly decompressed. The nerve root canal was explored along the nerve root, and expanded in the presence of stenosis (Fig. 3D).

#### 2.2.3. AUSS for bone anchoring annular repair technique

**Figure 4.**
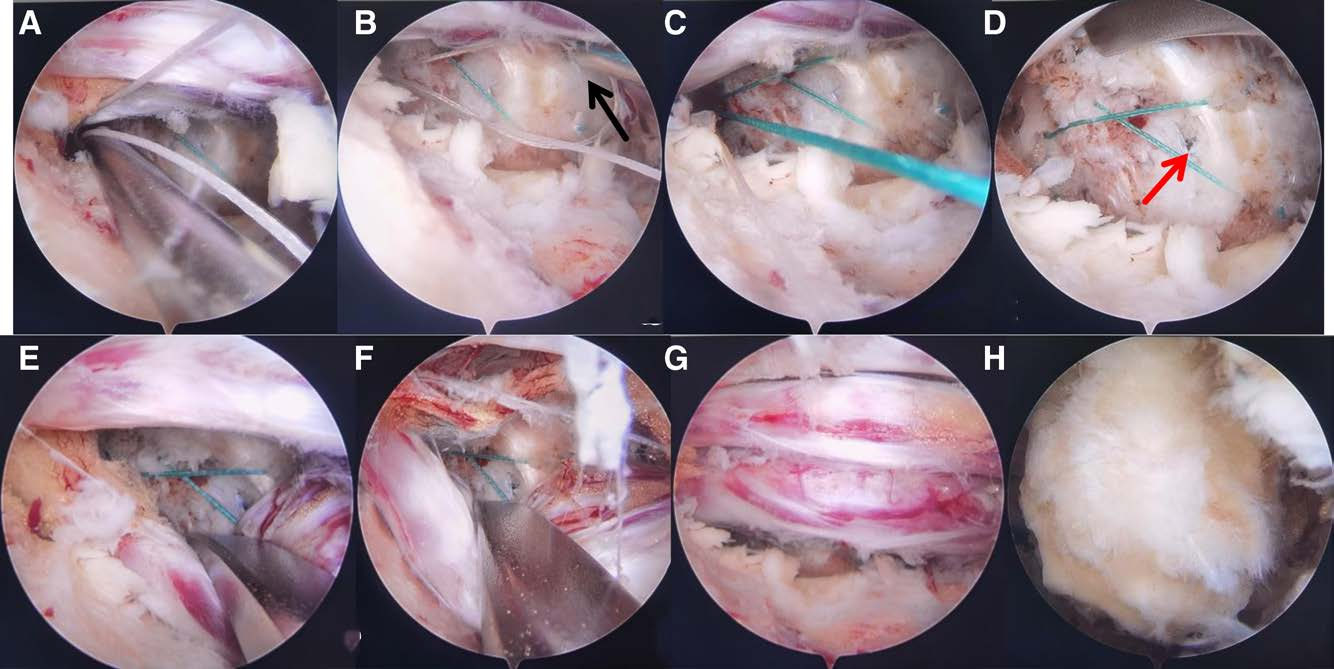
Operative illustrations in arthroscopic view. (A) A coiled suture needle was inserted located 3 mm laterally from the puncture point of the initial annular fibrous crossing. (B) Another Smile suture needle was inserted into the coil and punctured into the alternate hole of the vertebral body. (C) The first knot was automatically tightened. (D) The tear in the annular attachment area at the edge of the vertebra was sutured. (E, F) The postoperative effect of annular suture with bone anchoring. (G) The patient presented a lumbosacral nerve root anomaly (Neidre and Macnab’s classification Type-II). (H) The ligament flavum was returned to its original position to cover the epidural space.

After the removal of the protruding nucleus pulposus, a rupture in the annular attachment area at the vertebral body edge was observed (Fig. 3E). Subsequently, 2 holes were drilled parallel to the vertebral body, positioned 4 mm from the edge of the ruptured area, with a 3-mm spacing between them (Fig. 3F).The Smile suture needle was then inserted into the vertebral body through 1 hole, the thread rod was pushed in, and the suture needle was pulled out. In vitro, the coil on another suture needle with a coil was sheathed into the suture that had been sewn into the vertebral body. Under arthroscopic supervision, the suture needle was punctured into a position 2 mm away from the fracture site on the annular side. Subsequently, a thread rod was inserted before removing the suture needle, allowing the knot to slide in and lock automatically. Two additional knots were tied, and a knot pusher was used from the outside to the inside. The sutures were then cut using a suture cutter (Figs. 3G–L). Another Smile suture needle was inserted into another hole in the vertebral body, and the threaded rod was pushed into the body in a similar manner. The suture needle was pulled out, and a coil suture needle was introduced 3 mm lateral to the first annular puncture point. The initial knot tightened automatically, followed by the successive tightening of 2 more knots from the outside to the inside. Excess sutures were then cut off (Figs. 4A–D). This technique is referred to as the “bone anchoring” annular suture technique (Figs. 4E–G). Subsequently, the LF was returned to its original position, effectively covering the epidural space (Fig. 4H).

#### 2.2.4. Drainage and closure

A drainage tube was inserted into the incision, and the incision was closed with a silk thread. The estimated blood loss throughout the operation was 10 mL, and total operation time was 45 minutes.

## 3. Results

**Figure 5.**
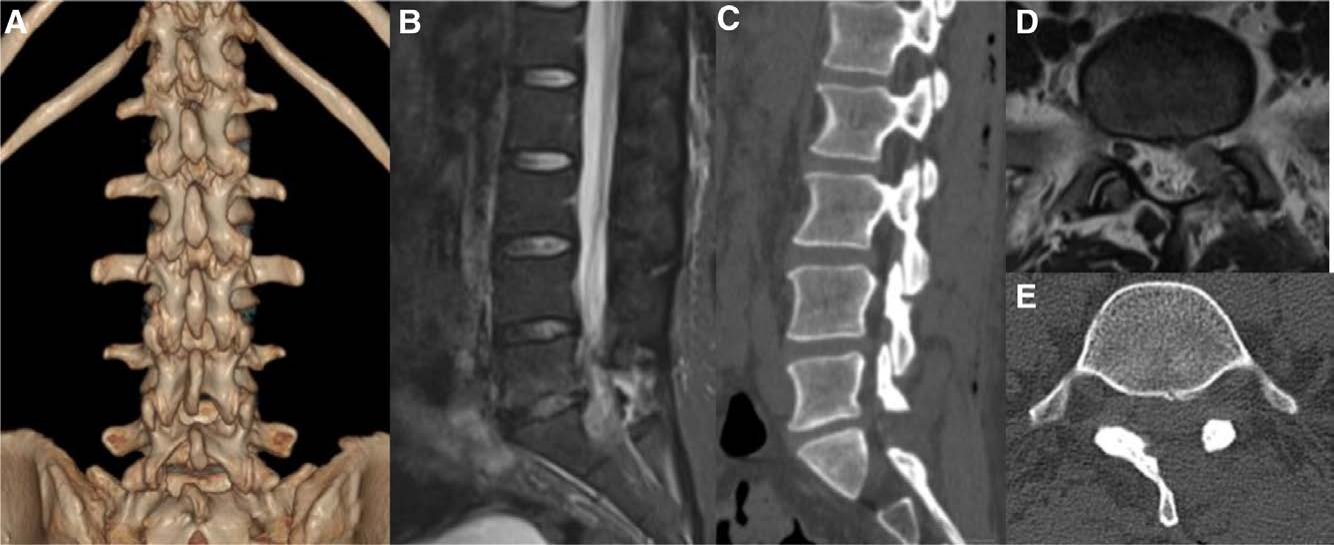
Patient postoperative radiologic images. (A) Three-dimensional reconstruction CT indicating the location of the L5-S1 laminectomy on the left side. (B–E) Postoperative MRI and CT images show that the protruding nucleus pulposus was successfully removed, and the spinal cord and nerve roots were completely decompressed.

There were no postoperative complications such as cerebrospinal fluid leakage and decreased muscle strength. The patient experienced a swift recovery, initiating postoperative ambulation after the drainage tube was removed 48 hours post-surgery. The lower limb pain and numbness was significantly improved, and the postoperative visual analogue scale score of the lower limb was reduced to 1. The postoperative Japanese Orthopaedic Association score reached 25, and the recovery rate was 76.5%. Postoperative X-ray demonstrated no signs of instability, CT and MR images affirmed the successful removal of the protruding nucleus pulposus and complete decompression of the spinal cord and nerve roots (Fig. 5).

## 4. Discussion

To the best of our knowledge, this study represents the inaugural application of AUSS, LF preservation, and bone anchoring annular suture technique in LDH treatment. With the assistance of arthroscopy, we successfully sutured the tear in the area where the annulus was attached to the edge of the vertebral body, effectively preventing LDH recurrence and significantly improving patient clinical symptoms.

With the continual evolution of minimally invasive concepts and technologies, endoscopic spinal surgery has gradually supplanted open surgery as the prevailing treatment for LDH. It offers advantages such as smaller incision, shorter operation time, reduced intraoperative blood loss, preservation of intact paraspinal soft tissue structure, lower complication rates, accelerated postoperative healing, and improved spinal stability. This approach has demonstrated remarkable efficacy in clinical practice.^[11–13]^ However, the uniaxial endoscopic technique has its challenges, including steep learning curve, limited visual field, constrained intraoperative operation space, inadequate exploration and decompression range, low surgical efficiency, residual nucleus pulposus tissue, and potential for symptom recurrence.^[14–16]^ In 2021, Professor Song En proposed the AUSS technology concept. Introducing the AUSS technique as a novel, minimally invasive spinal surgery, it uses a single surgical incision, allowing the surgeon to hold the arthroscope in one hand while operating the RF and surgical instruments with the other. With a large operation space and freedom, the AUSS technique addresses the limitations of the uniaxial endoscopic technique and reduces tissue damage compared with the unilateral biportal endoscopy technique. The high-definition imaging system of the arthroscope provides a clear and comprehensive view of the spinal cord and nerve structure, epidural venous plexus, and micro arteries. While sufficiently decompressing, it is conducive to hemostasis and significantly reduce the risk of nerve injury.^[17,18]^ AUSS technique can be performed with traditional posterior decompression surgical instruments of spinal surgery, ensuring operational efficiency while achieving a clinical decompression effect comparable with that of posterior open surgery.^[19]^ Moreover, the AUSS technique aligns with the principle of traditional fenestration discectomy surgery, making it more accessible for surgeons familiar with open surgery in spinal surgery. Consequently, the learning curve of the AUSS technique is relatively gentle.^[20]^

Percutaneous endoscopic discectomy is a commonly employed treatment for LDH. However, the postoperative recurrence rate of LDH is 5% to 19%,^[5]^ and the reoperation rate is 4% to 15%.^[6]^ Preserving the LF establishes a natural barrier between the paraspinal muscle and dural sac, reducing the formation of epidural scars. This preservation creates a safer anatomical plane for surgeons during reoperation, facilitating the location of anatomical structures and nerve tissues. It aids in retaining the original anatomical plane and reducing the formation of postoperative adhesions.^[7,8,21]^ Epidural adhesion or scar tissue hyperplasia is a primary cause of dural tears and nerve injury in recurrent disc surgery.^[22]^ Preserving the LF in the initial operation effectively mitigates operative time, surgical hemorrhage, and iatrogenic complications such as nerve root injury and cerebrospinal fluid leakage following a dural tear. This preservation strategy contributes to favorable outcomes for recurrent LDH surgery.^[8,23]^

After discectomy, a subset of patients continues to experience recurrent pain in the lower back and lower limbs.^[24]^ The incidence of failed back surgery syndrome attributed to postoperative fibrosis can be as high as 30%.^[25]^ Once fibrosis replaces the normal epidural tissue, it can impede the movement of the dura mater and nerve roots, leading to nerve root compression and recurrent pain.^[8,24,25]^ Fibrosis also induces epidural compression through the mass effect and nerve root ischemia via traction on the epidural region.^[26]^ Prevention or inhibition of epidural fibrosis is crucial for reducing recurrent lower back and leg pain.^[7]^ The LF serves as a natural barrier, and its preservation is essential to limit the degree of postoperative epidural fibrosis. We believe that safeguarding the LF, as the most secure and effective method, is crucial in reducing the extent of epidural fibrosis.^[27,28]^ Therefore, the LF preservation technique was adopted in this study.

Upon removal of the nucleus pulposus, if the ruptured annulus is not adequately closed, the residual nucleus pulposus may protrude again from the original annular tear when subjected to force.^[29]^ Furthermore, inadequate closure of the annular tear following nucleus pulposus excision may result in the release of inflammatory factors by the residual nucleus pulposus, stimulating the lower back nerves and causing persistent pain.^[10]^ Therefore, repairing the annular tear following nucleus pulposus excision is essential to effectively restore annular integrity, prevent residual nucleus pulposus protrusion, and reduce recurrence and reoperation rate.^[9]^ This approach also diminishes the exudation and release of inflammatory factors, alleviates nerve root stimulation by disc inflammation, mitigates postoperative lower back pain symptoms, and enhances patient quality of life.^[10]^ Furthermore, it reinforces the mechanical resistance of the intervertebral disc, averting degeneration caused by excessive nucleus pulposus excision while maintaining biomechanical strength and lumbar spine stability.^[30]^

LDH manifests in diverse forms, including herniation, sequestration-type herniation, calcified herniation, and extreme lateral type herniation.^[31]^ If the nucleus pulposus or endplate tissues protrudes, the location of annular tears also varies, which poses various challenges for the annular suture technique. Tears at the annular attachment area at the edge of the vertebral body (cephalad or caudad) are considered a prohibitively challenging area for annular suturing. Inspired by the “footprint area” concept in rotator cuff injury repair,^[32]^ we introduce the concept of “plane healing of footprint area in vertebral edge annular attachment area tear” for the first time. According to this concept, achieving full contact and closure between the “vertebral bone-annular attachment area” plane requires a multi-needle suture technique to facilitate prompt healing and prevent recurrence. The use of a double-needle X-shaped cross suture enhances the contact area between the annular and vertebral bone, promoting healing at the “bone-annular” interface. During surgery, a Smile suture device and a high-strength thread are used to increase compression in both the annular and vertebral edge footprint areas, serving as a barrier against residual nucleus pulposus in the early postoperative stages.

## 5. Conclusion

In conclusion, AUSS combined with LF preservation and the bone anchoring annular suture technique provides favorable clinical and imaging outcomes and is a safe and effective technique for the treatment of LDH.

## Author contributions

**Conceptualization:** En Song.

**Data curation:** Gushang Xia.

**Investigation:** Gushang Xia, Qingli Kong, Xianglin Li.

**Methodology:** Gushang Xia, Qingli Kong, Xianglin Li.

**Writing – original draft:** Gushang Xia.

**Writing – review & editing:** Gushang Xia, En Song, Qingli Kong, Xianglin Li
